# Bowtie Nanoantenna Coupled Metal-Oxide-Silicon (p-Doped) Diode for 28.3 THz IR Rectification

**DOI:** 10.3390/nano12223940

**Published:** 2022-11-09

**Authors:** Nasim Al Islam, Sangjo Choi

**Affiliations:** 1 Department of Electrical, Electronic, and Computer Engineering, University of Ulsan, Ulsan 44610, Korea; 2 School of Electronics Engineering, Kyungpook National University, Daegu 41566, Korea

**Keywords:** THz energy harvesting, MOS diode, plasmonic nanoantenna, low-temperature waste heat

## Abstract

Low-temperature waste heat in the infrared (IR) wavelength region offers an opportunity to harvest power from waste energy and requires further investigation in order to find efficient conversion techniques. Although grating-coupled metal-oxide-semiconductor (MOS) diode devices offer efficient conversion from low and moderate-temperature thermal sources, the integration of such diodes with a nanoantenna structure has yet to be explored. We propose a bowtie nanoantenna coupled with a p-doped MOS diode for IR to direct current (DC) conversion without any bias voltage at 28.3 THz. The nanoantenna was designed and optimized to provide maximum field enhancement in a 4 nm-thick oxide layer at the resonant frequency. The device was fabricated following the complementary MOS (CMOS) fabrication process and measured in a custom DC and optical characterization setup using a 10.6 μm wavelength CO_2_ laser. The results reveal two different types of devices with linear and nonlinear I-V curves having kΩ and MΩ zero-bias resistance, respectively. The linear device generates a micron-level open-circuit voltage (V_oc_) with clear polarization dependence from the laser input, but the nonlinear case suffers from a weak noise-like signal. Finally, we analyze two types of devices using thermoelectric and tunneling effects and discuss the future direction of nanoantenna-integrated MOS devices for efficient IR harvesters.

## 1. Introduction

More than 50% of the heat energy emitted from transportation, industrial machinery, and various other sources in a wide range of temperatures from −23 °C to 1200 °C remains unused and exists in the infrared (IR) frequency range of 20–100 THz [1,2]. For efficient global energy reuse, low-temperature waste heat must be utilized through efficient energy-harvesting techniques. Among existing methods, thermoelectric, thermophotovoltaic (TPV), and IR rectenna devices are commonly used for heat-to-energy conversion [3,4,5]. However, thermoelectric and TPV devices still face challenges in generating sufficient electrons above the noise level from low-temperature heat sources (≤230 °C) for real-world applications [6,7]. Thermal rectenna-integrated tunnel diodes address this limitation by rectifying a small-signal IR wave into direct current (DC) photocurrent through direct tunneling [5]. Efficient rectification in the tunnel barrier occurs when the photonic or plasmonic resonance of the antenna overlaps with the phonon resonance of the oxide layer near epsilon near zero dispersion region [8]. Multiple research groups have demonstrated direct tunneling through metal-insulator-metal (MIM), metal-double insulator-metal (MIIM), and metal-oxide-semiconductor (MOS) tunnel diode rectifiers [5,8,9,10,11,12,13,14].

Recently, several studies on MIM diodes have attempted to harvest energy from a 10.6 μm (28.3 THz) IR wave source, which is equivalent to a temperature of 200 °C [9,12]. When a MIM diode is illuminated by IR radiation, electrons tunnel through the oxide barrier between two metals with a high work function difference and generate a tunneling current. However, these single-insulator MIM diodes face the limitation of high resistance with low diode asymmetry, and, therefore, require further improvement. Progress has been made to increase the diode asymmetry by introducing an extra insulator layer between two metals via MIIM diodes [10,13,15]. Energy band barrier bending at the metal-insulator interface can be controlled using two different oxide materials to create a low-resistivity path for more electrons to tunnel through. The formation of such a path to increase the tunneling current depends on the precise control of the oxide layer thickness. However, controlling these oxide layer thicknesses below 2 nm levels with sufficient precision is challenging [10,16]. As an alternative to MIM diodes, IR rectenna devices integrated with MOS tunnel diodes offer a viable solution by utilizing complementary MOS (CMOS) fabrication techniques for ease of fabrication. Previously reported unipolar MOS tunnel diodes integrated with metal gratings achieved tunneling rectification through photon-assisted tunneling between the metal and semiconductor junction [5,8,11]. Further improvement in the IR-to-DC conversion efficiency was achieved using a bipolar MOS tunnel diode [17]. The existing MOS tunnel diodes use metallic grating structures for electrodes, similar to solar cells. Since nanoantennas have been used for high field enhancement and the impedance matching with a diode to increase the output of IR harvesters, integration of the nanoantenna into a MOS diode is worth exploring [18,19,20].

In this paper, we propose a nanoantenna-integrated p-doped MOS diode for low-temperature IR energy-harvesting applications. We utilized a bowtie-shaped nanoantenna and optimized the dimensions for maximum field enhancement inside the oxide barrier over p-doped silicon at 10.6 μm (28.3 THz). Next, we fabricated a nanoantenna-coupled MOS diode using standard CMOS fabrication process and demonstrated its performance in DC and IR measurement setups. We examined two different types of devices with linear and nonlinear I-V curves in the same batch and analyzed their working mechanisms. Using the IR measurement setup, we measured an open-circuit voltage (V_oc_) of both types by varying the polarization angle of the incident wave and found that the device with the nonlinear I-V curve subject to the tunneling effect failed to maintain a sufficiently high V_oc_ compared to the noise level. However, the other linear-type device exhibited a cosine-shaped micron level V_oc_, and we analyzed that the rectification mechanism was the thermoelectric effect. Finally, we discuss future research directions for the nanoantenna-integrated MOS diode devices for efficient IR to DC rectification.

## 2. Design Geometry and Simulation Method

**Figure 1:**
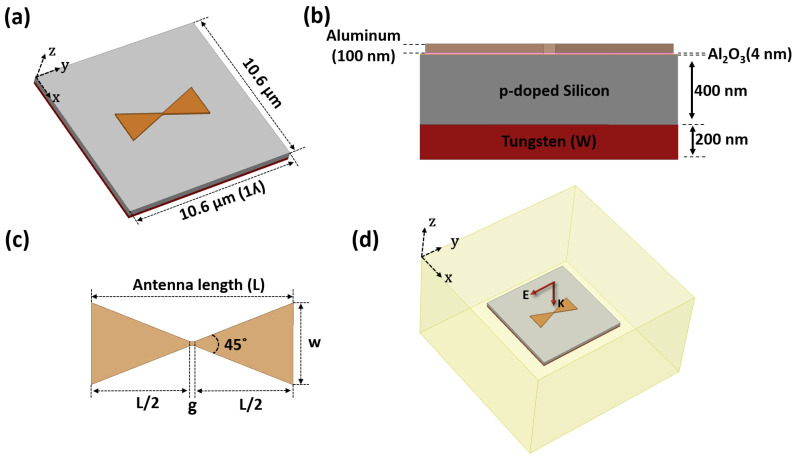
(**a**) The simulation model geometry of the nanoantenna-coupled p-doped MOS tunnel diode. (**b**) A cut-view of device geometry with layer thicknesses. (**c**) The detailed dimensions of the bowtie shaped nanoantenna. (**d**) The simulation environment inside a radiation boundary condition with a plane wave incident at 28.3 THz.

Figure 1a,b shows a simulation model and cut-view of the nanoantenna-coupled MOS diode geometry. A bowtie-shaped nanoantenna with connected arms is used as the antenna structure based on aluminum (Al). A thin layer (4 nm) of aluminum oxide (Al_2_O_3_) is used as an insulator layer between the metal (antenna) and a p-doped semiconductor (silicon) layer. Al_2_O_3_ was chosen as the oxide layer because its longitudinal optical (LO) phonon resonance is near the target wavelength, 10.6 μm. The match between the phonon and antenna resonance is intended for maximum field enhancement at the antenna from the near zero permittivity (*ε* = ~0) of the oxide material [8]. As a back reflector, a tungsten ground layer is used below the doped silicon layer to improve in-phase coupling between the incident and reflected waves near the antenna.

In this study, we used the finite element-based electromagnetic solver, ANSYS HFSS, to design and optimize the simulation model of the proposed nanoantenna-integrated MOS diode under a plane wave incident at 28.3 THz. The Al-based nanoantenna structure was optimized by placing it over the MOS diode configuration.

First, we fixed the bowtie angle to 45° and thickness to 100 nm, and varied the antenna length (L) to obtain the maximum field concentration at the antenna edges. Figure 1c shows the geometry of the antenna used for this device. Instead of proposing an air gap between the two arms of the bowtie-shaped nanoantenna, we connected two bowtie antenna arms with a square-shaped junction to maintain the DC path generated by the IR input and set the length (g) of the junction to 100 nm. In the simulation, we used a substrate size similar to the device’s wavelength (10.6 μm) to reduce the computation time while maintaining antenna performance. Figure 1d shows the HFSS environment used for this simulation, where the antenna-integrated MOS diode is illuminated with a y-polarized plane wave propagating along the z-axis at 28.3 THz inside a radiation boundary. For proper modeling and simulation accuracy, the real part of relative permittivity ($\varepsilon_{real})$ and conductivity (σ) of materials in the far-IR region were calculated using Equations (1)–(3) from the Drude-model [21,22,23]:

(1) $$\varepsilon_{real}\left(\omega \right)=n^{2}-k^{2}$$

(2) $$\sigma \left(\omega \right)=\varepsilon_{0}\times \varepsilon_{im}\times \omega$$

(3) $$\varepsilon_{im}\left(\omega \right)=2\times n\times k$$

### 2.1. Antenna Length Optimization for Maximum Field Enhancement

**Figure 2:**
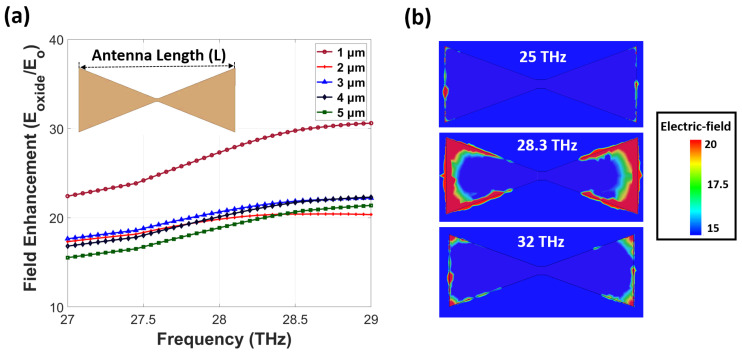
(**a**) The averaged field enhancement at 28.3 THz with variation of the antenna length at the oxide tunnel barrier below antenna edges and (**b**) cross-sectional view of field enhancement on the top of the oxide layer on the x-y plane for different frequencies, 25, 28.3, and 32 THz.

To ensure maximum enhancement in the oxide barrier, we optimized the antenna length and width for resonance at 28.3 THz. The field enhancement is defined as the ratio between the magnitude of the electric field at the measurement point and the incident electric field (E/E_0_). Here, E_0_ means the y-polarized plane wave with a field intensity of 1 V/m. To calculate the field enhancement level accurately, we placed four Al_2_O_3_ boxes with 5 nm × 5 nm × 4 nm dimensions under the four antenna edges with small mesh sizes and obtained the average field enhancement value. Figure 2a shows the averaged field enhancement inside the oxide layer below the antenna edges as a function of the antenna length (L). We found a maximum field enhancement of 29 for a 1 μm-long antenna with lower, but almost similar, values from 2–5 μm-long ones. We then selected the shortest length of 2 μm longer than 1 μm to secure the antenna pattern more accurately during the lithography process and simultaneously reduce deposition time and expense. Figure 2b shows a cross-sectional view of the field enhancement on the top of the thin oxide layer below the 2 μm-long antenna at different resonant frequencies, 25, 28.3, and 32 THz. Maximum enhancement is observed at the antenna edges at 28.3 THz and there is a reduction of the concentrated field when moving further from the resonant frequency.

### 2.2. Connecting Wire Length Optimization

**Figure 3:**
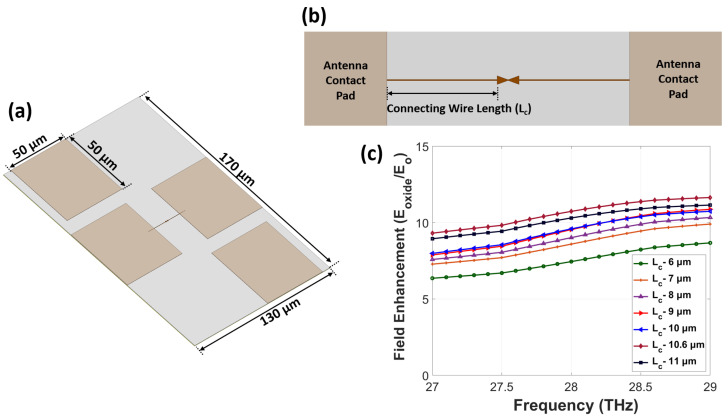
(**a**) The geometry of a nanoantenna-coupled p-doped MOS diode device with measurement pads. (**b**) The bowtie-shaped nanoantenna connected with the measurement pads through connecting metallic wires. (**c**) The frequency-dependent field enhancement versus length of the connecting wire (L_c_).

Figure 3a shows the geometry of the nanoantenna-integrated MOS diode with measurement pads. We used four different pads of size 50 μm × 50 μm to connect the antenna and p-doped silicon. The overall size of a single device was 130 μm × 170 μm. After fixing the antenna geometry, we tuned the lengths of the connecting metallic wires (L_c_) between the nanoantenna and measurement pads, which were used for mounting the probes. Here, the width of the wire was set to be 100 nm. The centers of the sides of the two antenna arms were connected to two metallic pads, as shown in Figure 3b. The measurement pads on the upper left and lower right corners were connected to the p-doped silicon layer through via connection. To ensure maximum field enhancement with a minimum effect from the connecting wire, we optimized L_c_. Figure 3c shows the field enhancement variation for different values of L_c_. As we varied L_c_ from 6 to 11 μm, we observed the maximum enhancement from a 10.6 μm (1λ)-long wire due to in-phase excitation between the two ends of the connecting wires.

## 3. Device Fabrication

The fabrication of the nanoantenna-coupled MOS tunnel diode was carried out by using the standard CMOS process. The process started with a p-type <100> 100 mm Si wafer. To remove the surface roughness created by native oxides over the Si wafer, buffered oxide etching (BOE) was performed. After etching, a 200 nm tungsten (W) ground layer was deposited by DC sputtering and a 400 nm poly-silicon layer was deposited over the ground layer using low pressure chemical vapor deposition (LPCVD) process. E-beam lithography was then used to pattern the p-type doping area with perfect alignment and to cover the remaining area with an e-beam resist to prevent dopant diffusion. Single-stage heavy boron implantation was then performed for p-type doping with a dose of 6.4 × 10^14^ cm^−2^ at 70 keV energy. Thermal annealing at 1000 °C for 10 s was carried out to activate and spread the dopants inside the poly-silicon layer. A very thin (4 nm) Al_2_O_3_ tunnel oxide layer was then deposited using atomic layer deposition (ALD) to ensure uniform distribution over the surface. Following the deposition process, the sample was coated with an e-beam resist and a bowtie-shaped nanoantenna, including connecting wires, and was patterned through an e-beam lithography lift-off process. With perfect position and alignment, a 10 nm-thick Ti adhesion layer and a 100 nm-thick Al antenna layer were deposited using the e-beam evaporation process. Before the deposition of four measurement pads, via holes with a diameter of 5 μm were etched in the p-doped silicon layer with a depth of 50 nm to ensure proper connection between the measurement pad and p-doped layer using inductively coupled plasma reactive ion etching (ICP-RIE). Finally, 50 μm × 50 μm contact pads were patterned through photolithography process and a 200 nm-thick Ti layer was deposited through the thermal evaporation. The fully processed four-terminal devices were imaged using a DM 4000M optical microscope (Leica Microsystems, Wetzlar, Germany) and a SU8220 scanning electron microscopy (SEM) (Hitachi High-Technologies, Tokyo, Japan).

**Figure 4:**
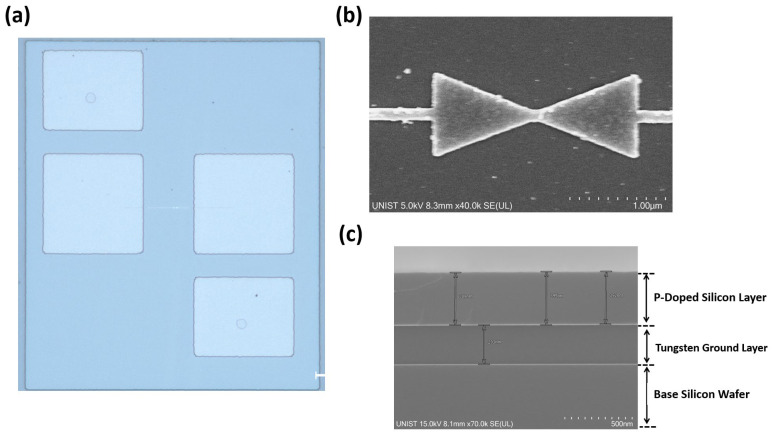
(**a**) The top view of fabricated device from an optical microscope with four measurement pads for DC and IR measurements. (**b**) The top view scanning electron microscopy (SEM) image of nanoantenna. (**c**) SEM image of cross-section of fabricated device.

Figure 4a shows the fabricated MOS device with four different measurement pads consisting of separate connections from the nanoantenna and p-doped semiconductor. The four terminals of the device offer flexibility in measuring the device in both two and four-probe measurement setups by applying a bias voltage. Among four terminals, two metallic pads were connected to the antenna arms through connecting wires for verification of antenna connection and I-V curve measurements. The measurement pads on the upper left and bottom right were connected through via holes to the highly doped p-type silicon layer. Figure 4b,c shows a top-view SEM image of the fabricated antenna and a cross-sectional SEM image of the device, where a 2 μm-long bowtie antenna shape with the connecting wires and the precisely controlled thicknesses of the oxide, semiconductor, and ground layer can be seen.

## 4. Results and Discussion

**Figure 5:**
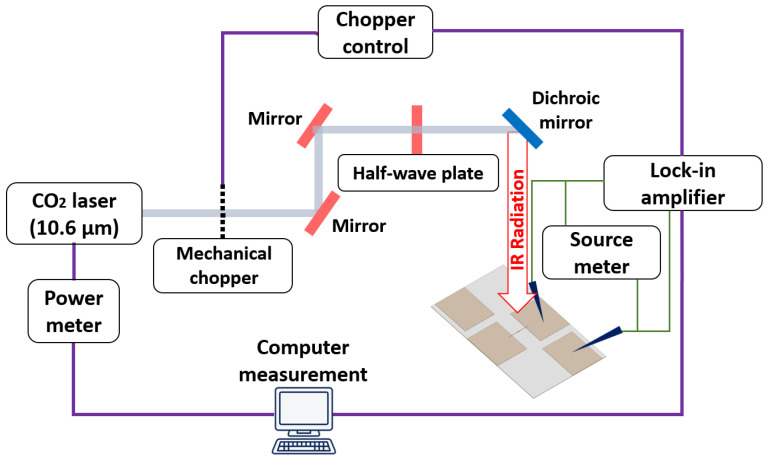
Schematic of the measurement setup.

We verified the performance of the nanoantenna-integrated MOS diode using DC and IR measurements. Figure 5 shows a schematic of the measurement setup. In the DC measurement setup, a high precision source meter KEITHLEY 2601B (Keithley Instruments, Cleveland, OH, USA) was used to accurately measure the current-voltage (I-V) curve between three different (antenna-antenna, semiconductor-semiconductor, and antenna-semiconductor) terminal pairs. The DC resistance between the terminals was calculated using the slope of the I-V curve (R = dV/dI). First, we verified the antenna connection of the device between the two measurement pads connected to the bowtie antenna arms. We found a low antenna resistance of approximately 80 Ω for both devices, indicating a good connection between the antenna and the measurement pads. We then measured the resistance between the other two measurement pads on the upper left and lower right corners where the p-doped silicon layer is connected through the vias. Approximately 300 Ω of the resistance verified a proper via connection.

**Figure 6:**
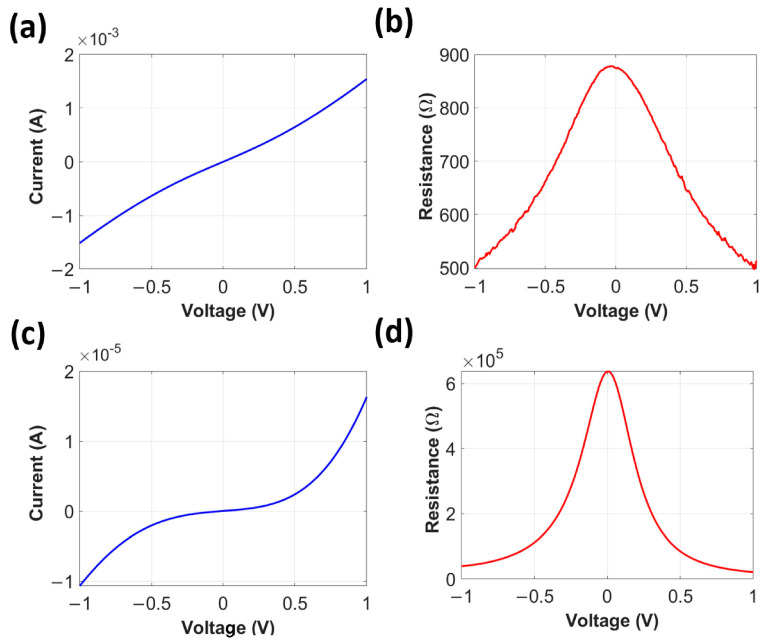
(**a**,**c**) Diode I-V curve and (**b**,**d**) differential diode resistance (R_d_) measurement from the linear and nonlinear devices, respectively.

Thirdly, we measured the diode’s I-V curve between two measurement pads connected to the antenna and p-doped semiconductor following the method reported for MOS diodes for IR harvesting [8,11]. Since the proposed device has four measurement pads, we can measure the diode performance from any of the two pads either from the left or right side, because the device is symmetric. Here, we report all the results measured on the right-side. From the measurements, we observed two different types of devices and reported the measured I-V curves and associated differential diode resistance for both types, respectively, in Figure 6a–d. Figure 6a,b shows an almost linear I-V curve with a low diode resistance (R_d_) of 890 Ω at zero bias from the first type of device. By contrast, we observed a highly nonlinear I-V curve pattern from the second type with a higher zero bias R_d_ of 0.6 MΩ, as shown in Figure 6c,d. From now, we will call the first and second types of devices “the linear device” and “the nonlinear device” despite a slight degree of nonlinearity in the first device, for convenience. In design geometry and fabrication condition, the linear and nonlinear devices do not have any difference. Therefore, the I-V curve difference may come from the fabrication uncertainty.

First, we understand that the linear device should have a dominant resistive path between the antenna and semiconductor layer due to a low zero bias R_d_ of 890 Ω. Composition variation or defects inside the 4 nm-thin Al_2_O_3_ layer can be the sources of the resistive path as reported from a thermoelectric effect-based rectenna device [24]. Second, the nonlinear device follows a tunnel diode-like I-V curve with a higher zero bias R_d_ of 0.6 MΩ. We expect that in the nonlinear device, the Al_2_O_3_ layer works as a tunnel barrier. The MΩ range of the measured R_d_ is higher than the kΩ range reported for MOS devices, and the reasons may be a smaller tunneling area of a single antenna and different semiconductor properties from the proposed device compared to previously reported unipolar and bipolar MOS devices with the electrically large area of multiple gratings [11,17].

**Figure 7:**
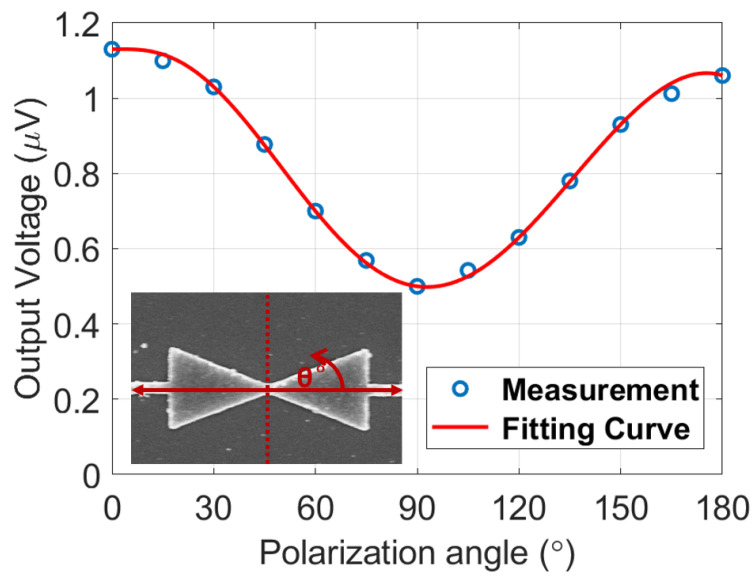
Polarization-dependent output voltage (V_oc_) from the device with the linear I-V curve.

Next, the IR performance of the nanoantenna-coupled MOS tunnel diode devices was measured at 28.3 THz at zero bias condition to check the rectification ability of the devices. During the IR measurements, the devices were illuminated with a linearly polarized CO_2_ laser operating at 10.6 μm (28.3 THz) with a power density of 1.77 W/cm^2^ and the polarization state of the electric field (E_0_) was set parallel to the antenna axis. The laser beam was modulated using a mechanical chopper with a frequency of 1 kHz and the laser beam was passed through a half-wave plate (HWP) to ensure proper polarization alignment with the antenna axis. Finally, the V_oc_ response of the device was measured using a SR830 DSP lock-in amplifier synchronized with the chopper. We measured the V_oc_ of the linear device and the value was 1.1 μV for 0° polarization angle of the incident wave. We also measured the polarization-dependent V_oc_ from the device and confirmed that a maximum V_oc_ of 1.1 μV is maintained when the polarization angles of the illuminating beam (0° and 180°) were aligned with the antenna axis. Conversely, a 90° polarization angle perpendicular to the antenna axis results in a minimum V_oc_ of 0.5 μV. The polarization-dependent nature of the linear device is shown in Figure 7, demonstrating the proper nanoantenna functionality of the device.

The polarization dependence and reasonably high V_oc_ level of 1.1 μV of the linear device compared to the reported nanoantenna-based MIM and thermoelectric devices are noteworthy [9,14,25,26]. We analyze that the high V_oc_, despite the kΩ level linear resistance of the device, is only possible by thermoelectric effect based on the similar trend reported from thermoelectric IR harvesting devices [24,27]. The heated metallic antenna area on the top layer owing to the laser input and the relatively cold semiconductor layer can maintain a temperature difference (ΔT) for the thermoelectric effect. From the Seebeck coefficients of Al and p-doped Si (S_Al_ = 3.5 μV/K and S_p-doped Si_ = 170 μV/K), the V_oc_ of 1.1 μV can be translated into a ΔT of 6.5 mK using the Equation (4) [28].

(4) $$V_{\mathrm{oc}}=\Delta S\times \Delta T=\left({\;S}_{p-\mathrm{doped}\;\mathrm{Si}}-S_{\mathrm{Al}}\right)\times \Delta T$$

We also note a non-negligible V_oc_ level of 0.5 μV at a 90° polarization angle and expect that it originates from the resistive connections spread inside the oxide. Although the input polarization is not aligned with the antenna axis, the heated measurement metallic pads can also contribute to the thermoelectric effect through dominant Al composition or defects inside the thin Al_2_O_3_ layer under the pads. For the 0° polarization angle case, the antenna edges heat up more than at other angles, and a higher V_oc_ thus can be expected.

We then measured V_oc_ from the nonlinear device and observed a noisy V_oc_ signal, as judged by the unstable fluctuation of the signal phase in the lock-in amplifier. Thus, we were unable to measure the polarization-dependent V_oc_ of the nonlinear device. The unstable noisy V_oc_ from the nonlinear device can be explained by Johnson noise, which increases with the higher resistance of the device [24,29,30]. We understand that the nonlinear device maintains a weak tunneling current level that is manifested by a MΩ zero-bias resistance, and this level is below the Johnson noise. The other V_oc_ generation from the thermoelectric effect may not be possible due to the highly resistive (insulator-like) barrier between the metal and semiconductor layers of the nonlinear device. Overall, the proposed nanoantenna integrated with the p-doped silicon showed a low tunneling-based V_oc_ and we expect that the reasons are a smaller tunneling area from the single antenna and different quality of p-doped silicon layer compared to the other grating-based MOS devices [5,11]. In future work, if the nanoantennas are designed on a more efficient bipolar MOS configuration and are constructed in a massive array, then a higher V_oc_ with a polarization-dependent trend will be possible [17].

## 5. Conclusions

In this study, we developed an IR to DC rectification device at 28.3 THz by using a MOS diode coupled with a bowtie-shaped nanoantenna. We optimized the nanoantenna structure on a 4 nm-thick Al_2_O_3_ layer and p-type silicon grounded by a metal reflector to achieve maximum field enhancement at the antenna edges. For an optimum device, the lengths of connecting wires between the antenna and the measurement pads were also optimized. Using the optimized geometric parameters of the nanoantenna, we fabricated the device by using a standard CMOS fabrication process. We then measured the diode performance by varying the bias voltage and found two different device types with linear and nonlinear I-V curves. The linear device that shows near 1 kΩ at zero bias may be enabled by composition variation or defects in the thin Al_2_O_3_ layer, and we expect that the device is subject to the thermoelectric effect. By contrast, the nonlinear device maintained a resistance close to 1 MΩ, which could be due to weak tunneling. Optical measurements using a 10.6 μm CO_2_ laser confirm a higher open-circuit voltage (V_oc_) of 1.1 μV from the linear device with a clear polarization-dependent output. However, the nonlinear device did not produce a sufficiently high V_oc_ above the noise level. The small tunneling area of the single nanoantenna and different silicon properties compared to the electrically large grating-based MOS device may lead to weak tunneling. Despite the weak tunneling effect of the single nanoantenna, we expect that a massive array of nanoantennas with further engineering of the diode configuration can overcome the low V_oc_ limitation.

## Data Availability

Not applicable.
